# Updates in Motor Learning: Implications for Physical Therapist Practice and Education

**DOI:** 10.1093/ptj/pzab250

**Published:** 2021-10-25

**Authors:** Kristan A Leech, Ryan T Roemmich, James Gordon, Darcy S Reisman, Kendra M Cherry-Allen

**Affiliations:** 1 Division of Biokinesiology and Physical Therapy, University of Southern California, Los Angeles, California, USA; 2 Department of Physical Medicine and Rehabilitation, Johns Hopkins University, Baltimore, Maryland, USA; 3 Center for Movement Studies, Kennedy Krieger Institute, Baltimore, Maryland, USA; 4 Physical Therapy Department, University of Delaware, Newark, Delaware, USA

**Keywords:** Instructive, Motor Learning, Rehabilitation, Reinforcement, Sensorimotor Adaptation, Use-Dependent

## Abstract

Over the past 3 decades, the volume of human motor learning research has grown enormously. As such, the understanding of motor learning (ie, sustained change in motor behavior) has evolved. It has been learned that there are multiple mechanisms through which motor learning occurs, each with distinctive features. These mechanisms include use-dependent, instructive, reinforcement, and sensorimotor adaptation-based motor learning. It is now understood that these different motor learning mechanisms contribute in parallel or in isolation to drive desired changes in movement, and each mechanism is thought to be governed by distinct neural substrates. This expanded understanding of motor learning mechanisms has important implications for physical therapy. It has the potential to facilitate the development of new, more precise treatment approaches that physical therapists can leverage to improve human movement. This Perspective describes scientific advancements related to human motor learning mechanisms and discusses the practical implications of this work for physical therapist practice and education.

## Introduction

Promoting motor learning (ie, sustained change in a motor behavior) is a fundamental objective of many interventions within neurological physical therapy. Though this may be true now, it has not always been. Prevailing treatment philosophies (or “best practices”) in neurological physical therapy have evolved over time. Over the last 60 years, the predominant approach to treating patients with neurological damage or disease has shifted from mainly a neurophysiology-based approach to a more pragmatic and eclectic approach that emphasizes motor learning principles along with exercise science and biomechanics.^1^^,^^2^

This evolution was prompted by discoveries in the fields of behavioral motor learning and neuroscience and an increased awareness of the relevance of this work to the field of physical therapy.^3^^,^^4^ A detailed historical overview of the integration of motor learning research into neurological physical therapist practice is provided by Winstein et al.^5^ Recently, it has become evident that neuroscience and behavioral motor learning research are also relevant to other areas of physical therapist practice (eg, orthopedics^6–9^ and pelvic health^10^^,^^11^).

Accordingly, motor learning has become a fundamental component of entry-level physical therapy education. This includes content related to the inherent plasticity of the nervous system that can be driven through repeated practice of a new movement. Physical therapy curricula also commonly include behavioral motor learning research findings that provide guidelines on how to structure interventions to promote motor learning (eg, variable versus constant practice,^12^^,^^13^ feedback schedules,^14^^,^^15^ principles of behavioral economics^16^^,^^17^). Although this approach to teaching motor learning concepts is useful and an improvement over what was taught in previous years, it does not reflect more recent advances in motor learning research.

As physical therapists have begun to integrate principles of motor learning and neuroplasticity into practice and education, motor learning research has also continued to advance. We now have a more sophisticated and comprehensive understanding of *how* motor learning occurs. Specifically, we have learned that (1) there are multiple mechanisms of motor learning, (2) these mechanisms have distinct features, and (3) they primarily engage different areas of the nervous system. For an overview of these scientific developments, please see reviews by Krakauer and Mazzoni^18^ in 2011, and Kitago and Krakauer^19^ in 2013. Expanding our motor learning knowledge base to include these findings will allow physical therapists to diversify the means through which they can influence human movement. This is critical to the advancement of movement rehabilitation. Unfortunately, these discoveries have remained largely confined within the research community, despite their relevance to physical therapy (as discussed in a recent review by Roemmich and Bastian^20^).

To translate these scientific advances into evidence-based practice, we must first integrate them into our current understanding of motor learning. In this Perspective, we describe 4 well-studied mechanisms of human motor learning: use-dependent, instructive, reinforcement, and sensorimotor adaptation. Specifically, we outline the current explanatory framework for each of these motor learning mechanisms and we discuss the practical implications of each mechanism for physical therapist practice and education. Broadening our understanding of motor learning to include the multiple mechanisms through which it occurs will ultimately guide and sharpen physical therapist practice and promote future clinical and research innovation.

## Four Mechanisms of Motor Learning

**Figure 1:**
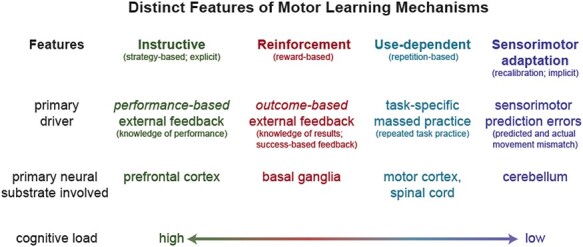
Key features of 4 distinct motor learning mechanisms. This represents a simplified summary of a large body of research aimed to understand how changes in human motor behavior occur. Alternative terms commonly used in the literature for these mechanisms and their features are provided in parentheses.

Through significant research efforts, we have come to understand that motor learning does not occur through a single process. Rather, there are multiple mechanisms of motor learning that can occur in parallel or in isolation to ultimately lead to sustained changes in motor behaviors.^19^^,^^21^ In this section, we provide an overview of 4 widely studied motor learning mechanisms: use-dependent, instructive, reinforcement, and sensorimotor adaptation-based motor learning. We will describe the key features of these mechanisms including the primary behavioral drivers, neural substrates, cognitive involvement, and timescales. Here we define the primary behavioral driver as the task demands that will increase the relative contribution of each learning mechanism to the overall change in behavior. This is perhaps the most clinically relevant feature of these motor learning mechanisms, because it is the element that physical therapists can manipulate through the structure of their interventions. The distinct features of each mechanism are summarized in Figure 1. In alignment with the motor learning literature, we use the term “mechanism” generally to refer to the processes through which motor learning occurs. This differs from its use in rehabilitation literature, in which it often describes a neurophysiological process underlying a clinical presentation or observed behavior. Of note, to help clarify how the concepts described here are related to existing literature, commonly used synonyms for various motor learning terms are provided in Figure 1.

The current state of science related to these motor learning mechanisms is still largely based on laboratory-based research. There have been few intervention studies (eg, Reisman et al^22^ and Wolf et al^23^) to inform the design of robust treatment protocols or the development of guidelines for clinical implementation. Therefore, we will primarily focus on the potential practical implications of these advances in motor learning research versus direct clinical application.

### Use-Dependent Motor Learning

Use-dependent motor learning (Fig. 1; blue) is defined as a change in motor behavior that is driven by repeated task-specific practice. In use-dependent learning, repeated practice of a new movement causes future repetitions to be more similar to the practiced behavior. For instance, a golfer repeatedly practices a new swing technique so that future attempts to produce the skilled behavior will result in the expression of the well-practiced movement pattern. This form of motor learning has been well studied and is widely accepted as a powerful treatment tool to promote changes in motor behavior. A large body of work has demonstrated that the primary behavioral driver of use-dependent motor learning is the amount of practice completed by the learner.^24^ Importantly, researchers have also identified a number of practice parameters that can be manipulated to optimize use-dependent learning (task specificity, aerobic intensity, etc.).^25^

Improvements in motor behavior resulting from repeated practice are mediated by structural and functional changes throughout the central nervous system^26–29^ (referred to as *experience-dependent neuroplasticity*). Thus, there is a natural overlap between the drivers of use-dependent motor learning and the canonical principles of experience-dependent neuroplasticity as described by Kleim and Jones^30^ in 2008—commonly taught as the guiding principles to optimize motor behavior change in many current doctor of physical therapy curricula.

Another important feature of use-dependent motor learning is the degree of cognitive processing that is required. Studies have demonstrated that passive or overly guided movement practice (eg, assisted passive range of motion, manual facilitation of a movement pattern) does not lead to changes in motor behavior^31^ or neuroplasticity.^32^ As Bernstein described, “practice … does not consist of repeating the means of solution of a motor problem time after time, but in the process of solving this problem again and again by techniques we have changed and perfected from repetition to repetition”; rather, “practice is a particular type of repetition without repetition.”^33^ For motor practice to drive use-dependent learning, the learner must be exerting motor effort to actively practice the task. In addition to active movement, use-dependent learning also requires a degree of cognitive engagement in the task—that is, the participant must understand the task goal and make intentional changes to their movements to solve the movement problem and achieve that goal. Ultimately, extensive task practice of this sort will automatize behaviors and reduce the cognitive load required to complete them.^34^

One of the disadvantages of use-dependent motor learning is that it occurs over a long timescale. This means that lasting improvements in motor behavior are slow to accumulate (ie, over weeks or months)^23^ and the generalization from training to real-world improvement can be quite small^35^ with interventions that primarily depend on use-dependent motor learning. One approach to elicit more immediate changes in motor behavior is to combine use-dependent learning in parallel with instructive motor learning, which occurs on a much faster timescale. This is discussed in more detail in the section below.

### Instructive Motor Learning

Instructive motor learning (Fig. 1; green) is defined as a change in motor behavior achieved through the use of an intentional movement strategy.^36^^,^^37^ This mechanism of motor learning is elicited when a learner is provided specific external feedback about a movement error or performance relative to a task goal (ie, knowledge of performance) that prompts the development of an intentional error-reducing movement strategy. An important component of instructive motor learning is that the error-reducing movement strategy can be explicitly described by the learner^38^ and reproduced in the future.^39^ In the context of physical therapy, the error-reducing movement strategy is often instructed or cued by a physical therapist (further described below). Therefore, here we refer to this mechanism as instructive motor learning, but it should be noted that it is also commonly termed explicit or strategy-based motor learning.^40^

Though not widely referred to as “instructive motor learning” in the clinic, this learning mechanism is a staple in rehabilitation interventions. Physical therapists often provide external feedback about undesirable movements (eg, verbal and/or visual cues about uneven step lengths during walking) and explicitly instruct corrective movement strategies (eg, “take a bigger step with your left leg”). Moreover, a majority of the investigators conducting early behavioral motor learning research related to physical therapy focused on understanding instructive learning. Many studies were conducted to explore the most effective modality (eg, auditory, visual, tactile) and timing of external feedback to provide to the learner.^41^ As such, instructive learning is a major focus of motor learning–related content in physical therapy curricula.

The prevailing theory is that instructive motor learning is largely mediated by structures in the prefrontal cortex^40^^,^^42^ and involves multiple cognitive processes (eg, comprehension of instructions, performance monitoring).^43^^,^^44^ This is supported by evidence that individuals with frontal lobe lesions and neuropsychological impairments have difficultly developing^38^ and retaining^36^ explicit strategies to correct undesirable movements. A recent study in individuals poststroke also demonstrates a strong relationship between cognition and the capacity for instructive motor learning.^45^ The high cognitive burden of instructive learning is likely intuitive to many clinicians; an external feedback-heavy treatment approach is often ineffective for improving motor behaviors in patients with cognitive deficits.

One key advantage of instructive motor learning is that it occurs on a relatively fast timescale when cognition is intact. When the correct movement strategy is executed, motor behavior can improve markedly within a single session or even from one repetition to the next. Importantly, movements acquired through instructive learning can be voluntarily recalled when the learner is prompted to use the newly learned movement strategy in the future.^39^ Retention, defined as the persistence of a behavior beyond the training period, is a hallmark of motor learning. This differentiates instructive learning-dependent improvements in motor behavior from transient changes in motor performance.^41^

Consider a patient poststroke who walks with reduced stance time on their weaker leg. A therapy session structured to make use of instructive motor learning (eg, external feedback about stance time symmetry) would enable this patient to learn a new movement strategy to walk with a longer stance time on their weaker leg by the end of the session. Experienced clinicians are aware that this patient will most likely return for the next visit exhibiting the same stance time asymmetry as they did prior to the last treatment session. From this, one may assume that the previously employed movement strategy to reduce stance time asymmetry was not retained. However, to assess retention of a learned movement strategy, the learner must be prompted or instructed to reproduce the learned behavior. If the patient can voluntarily produce a desired motor behavior after initial learning, then the instructive motor learning was retained. Importantly, whereas movement strategies can be retained and explicitly recalled in the future, the extensive practice that drives use-dependent learning is likely necessary for the new movement to become automatic or habitual.^20^^,^^34^ Development of a new movement habit may also require practicing within multiple contexts that *require* the patient to employ that movement strategy in order to be successful. However, identification of the key ingredients that drive a patient to transition from voluntary recall of a learned movement strategy to habitual use of that behavior remains a critically important goal of ongoing investigation.

### Reinforcement Motor Learning

Reinforcement motor learning (Fig. 1; red) is defined as an improvement in motor behavior that is driven by binary outcome-based feedback. That is, reinforcement learning depends on external feedback about the success or failure of the movement relative to a task goal (ie, knowledge of results). After a movement success or failure, the learner does not receive information about *how* the movement needs to be modified (or not) in order to be successful.^21^ This type of nondirectional external feedback prompts the learner to explore different movements and select actions that have the highest probability of success, while avoiding actions with low probability of success (reviewed in Sutton and Barto^46^ and Lee et al^47^).

The basal ganglia are thought to be the primary neural substrates involved in selecting movements that result in task success.^48^ This is potentially mediated by reward-based dopamine signaling, where activity of dopaminergic neurons increases in response to task success during early stages of practice.^49^ The primary motor cortex may also be involved in reinforcement learning, because recent evidence shows that reinforcement learning leads to plasticity in the primary motor cortex.^50^ This may be due to the connections between the basal ganglia and primary motor cortex within the basal ganglia-thalamo-cortical circuits.

The involvement of cognitive processes in reinforcement motor learning is unclear. Traditionally, it was thought that reinforcement learning was a more implicit or automatic process. One theory is that a successful movement would spontaneously occur during practice due to the inherent variability in the individual’s movement, and this would lead to an automatic biasing of future movement selection toward the successful or rewarding outcome.^46^ However, it now appears that intentional exploration of different movements in search of the successful behavior may be critical for reinforcement learning.^51^ Additionally, explicit control or development of an intentional strategy may also play an important role in reinforcement motor learning.^52^ From this emerging work, we suggest reinforcement motor learning may require more cognitive processing than originally proposed.

Reinforcement learning can lead to sustained behavioral improvements within a session. However, relative to sensorimotor-based adaptation (discussed below), these improvements take longer to develop.^53^ Despite the apparent slower rate of learning, reinforcement is associated with longer retention of acquired movements.^53^^,^^54^ This feature of reinforcement learning is particularly attractive for designing interventions with long-lasting effects. Recently, researchers have started to study how reinforcement-driven retention can be coupled with learning mechanisms that drive faster initial changes in motor behavior to be both quickly acquired and long-lasting. For instance, research groups that add success-based feedback to sensorimotor adaptation^55^^,^^56^ or protocols that require practice of a novel task^54^ have found improved retention relative to execution of those protocols without engaging reinforcement learning.

### Sensorimotor Adaptation-Based Motor Learning

Sensorimotor adaptation-based motor learning (also termed sensorimotor adaptation; Fig. 1; purple) is defined as a change in motor behavior that is driven by sensory prediction errors.^57^ Sensory prediction errors are perceived in the nervous system when the actual sensory consequence of a movement (most commonly detected by visual, auditory, or proprioceptive feedback) differs from the predicted sensory consequence of that movement. This most often occurs when individuals encounter unexpected task demands or changes in the environment (ie, perturbations) that require modifications to the executed motor program. When these errors are detected, the motor command is automatically updated to adapt the movement and reduce the magnitude of the prediction error (see Bastian^58^ for review). Imagine, for example, the experience of driving a car in which the brake sensitivity is different than your own. Applying a certain amount of pressure to the brake pedal will cause this car to stop more abruptly than anticipated. The perception of this sensory prediction error will prompt an adaption of the movement to reduce the pressure applied to the brake in the future, allowing you to decelerate the car smoothly. This is the same process that occurs, for instance, when a patient is learning to navigate a manual wheelchair over carpet versus tile or more generally when therapists vary the conditions of movement practice according to Gentile’s taxonomy of tasks.^59^ The phenomenon of sensorimotor adaptation aligns with the recognition schema proposed by Schmidt^60^ in 1975, and allows for an impressive degree of movement flexibility. It has been demonstrated across many types of movements including eye movements,^61^ arm movements,^62^ and walking.^63^

Our current understanding is that this mechanism of motor learning is primarily dependent on the cerebellum.^57^ The cerebellum is known to be involved in mapping outgoing motor commands to the predicted sensory feedback from that movement.^64^ Moreover, the ability to update motor commands and adapt movements in response to sensorimotor prediction errors is impaired in individuals with cerebellar disorders.^65–67^ Importantly, the capacity for sensorimotor adaptation-based learning may be related to the severity of cerebellar degeneration.^68^

Sensorimotor adaptation is thought to occur automatically and implicitly, independent of intentional, voluntary modifications to movements. This is demonstrated by evidence that sensorimotor adaptation still occurs even when people are instructed how to correct their movements,^69^ provided external error feedback,^70^ or prevented from correcting their movement errors.^71^ Interestingly, mounting evidence from studies of upper limb movements suggests sensorimotor adaptation often occurs in parallel with instructive or strategy-based motor learning.^40^ This indicates that the overall reduction in movement errors in many of the studied tasks may be due to simultaneous sensorimotor adaptation and development of explicit strategies to counteract an experimental perturbation. Evidence for the involvement of strategy-based learning during the adaptation of walking (a more continuous, patterned, automatic behavior) is emerging but less well established. In a study of locomotor adaptation the authors found that participants, when instructed, could voluntarily reproduce part of a new walking pattern that was learned through sensorimotor adaptation.^39^ This suggests that cognitive strategies may also contribute to locomotor adaptation, but more work is necessary to understand how these voluntary and involuntary motor learning mechanisms interact to elicit an overall change in behavior during walking.

Sensorimotor adaptation occurs on a very rapid timescale, generally resulting in a change in motor behavior over minutes. Movements are adjusted repetition-by-repetition to quickly reduce sensory prediction errors, allowing for an impressive flexibility of movement in the context of many different task demands.^58^ Importantly, these rapid adjustments are not simply transient changes in movement performance. Rather, the newly learned movement must actively be unlearned. That is, when the error-inducing perturbation is removed, participants will initially exhibit movement errors in the opposite direction until the adapted motor command is unlearned through the same error-reducing process (this behavioral phenomenon is called an “aftereffect”). There is also evidence that the adapted motor behavior is automatically stored for future use. When participants are reexposed to a perturbation, they (1) make smaller movement errors initially and (2) relearn the new movement faster.^72^ These behavioral phenomena are called “recall” and “savings,” respectively, and they are considered evidence of lasting motor memories formed through sensorimotor adaptation. Repeated exposure to a perturbation may ultimately lead to the establishment of a new, independent motor command,^73^ but the time course of this process across motor tasks is unclear.^74^^,^^75^

## Implications for Physical Therapist Practice and Education

We now appreciate that there are multiple, distinct, co-occurring motor learning mechanisms that subserve sustained changes in movement. This more comprehensive understanding of motor learning has powerful implications for physical therapy. Most importantly, it diversifies the methods that clinicians have at their disposal to promote motor learning. Physical therapist interventions across practice settings are currently structured to predominantly leverage use-dependent and instructive motor learning. Moving forward, physical therapists must begin also to consider reinforcement and sensorimotor adaptation as part of their arsenal. These 4 motor learning mechanisms can occur in parallel as a new movement is being learned, and the relative contribution of each can be manipulated by incorporating their unique drivers into movement practice. Physical therapist interventions can be carefully designed to leverage any or all of the 4 motor learning mechanisms described here.

**Figure 2:**
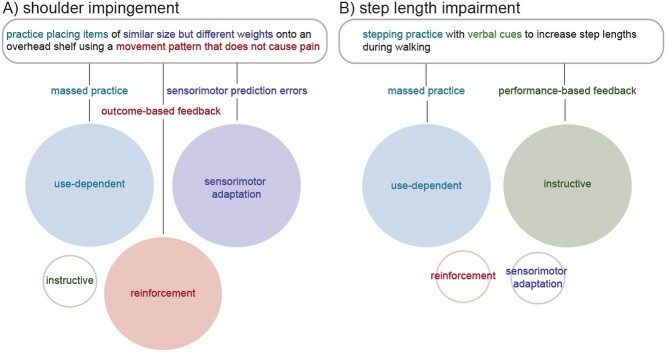
Motor learning mechanisms driven by example physical therapist interventions to treat shoulder impingement (A) and gait impairment (B). The relative contribution of each mechanism (represented by circle size and fill) to the targeted motor behavior change depends on the behavioral drivers that are integrated into the intervention by the physical therapist. Note that multiple mechanisms may often occur in parallel as a motor behavior is being learned.

This point is best illustrated with clinical examples. Consider a patient with shoulder impingement who has a goal to reach overhead without pain. To achieve this goal, a physical therapist has the patient practice placing items of similar size but different weights onto an overhead shelf using a movement pattern that does not cause pain. This intervention structure integrates 3 behavioral drivers that will promote different motor learning mechanisms to contribute to the overall change in reaching movement pattern (Fig. 2A): (1) repeated reaching practice will drive use-dependent motor learning, (2) if the patient considers a pain-free reach a successful outcome, using a movement pattern that does not cause pain would drive reinforcement motor learning, and (3) finally, practicing lifting objects of the same size but different weights will induce sensorimotor prediction errors that drive sensorimotor adaptation to promote flexibility of the pain-free reaching movement. With this intervention, the contribution of instructive motor learning is relatively small. If the patient has difficulty reaching overhead without pain or adapting the movement to account for objects of different weights, the therapist could consider modifying the intervention to include verbal cues for proper scapular dynamics during the reaching movement. This would increase the relative contribution of instructive learning.

Another example that demonstrates how physical therapists can carefully design interventions to target these learning mechanisms is a patient with Parkinson disease who has a goal of improving their gait speed. To achieve this goal, the therapist has the patient practice walking on a treadmill while providing verbal and visual cues to increase right and left step lengths (Fig. 2B). With this intervention, massed stepping practice will drive use-dependent motor learning, and the performance-based feedback from external cues about step lengths will engage instructive motor learning. To increase the contribution of sensorimotor adaption, the therapist could have the patient practice walking with longer step lengths over surfaces with variable levels of compliance that would include sensorimotor prediction errors (ie, the same propulsive force would lead to different step lengths relative to the initial practice conditions). Finally, in this case, the therapist would likely try to avoid an intervention structure that would rely on outcomes-based feedback, because attempting to leverage reinforcement motor learning in a patient with a pathology of the basal ganglia may be unsuccessful.

The multiplicity of motor learning mechanisms that contribute to sustained improvements in motor behavior is likely to influence practice in 2 main ways. First, prior to treatment the physical therapist can strategically design an intervention that accounts for individual patient characteristics and targets the motor learning mechanisms most likely to change motor behavior. For example, if a patient exhibits cognitive impairments, the physical therapist might structure the intervention to preferentially target motor learning mechanisms that require less cognitive processing (eg, sensorimotor adaptation). Another important patient characteristic to consider is the integrity of different areas of the nervous system, given that each mechanism of motor learning is primarily subserved by a distinct neural substrate. This is specifically important in the context of a patient with neurological damage or disease. For instance, a person with cerebellar degeneration may benefit from a physical therapist intervention that targets learning through reinforcement, instructive, or use-dependent motor learning rather than sensorimotor adaptation.

Secondly, having access to multiple mechanisms of motor learning may improve the therapist’s capacity to adjust interventions based on patient responsiveness throughout the course of treatment. Recent evidence demonstrates that there are interindividual differences in the way people learn new movements. One study found that approximately 20% of neurotypical participants were not able to learn a new upper extremity movement through reinforcement.^51^ This suggests that when a patient with any diagnosis exhibits a lack of responsiveness to an intervention, it may be useful to intentionally integrate another motor learning mechanism into the treatment approach. The ability to sharpen our clinical practice in these ways will have a significant impact on the physical therapist’s approach to movement rehabilitation across patient populations and areas of practice.

Research related to the motor learning mechanisms described here is progressing rapidly, and the field has clearly advanced beyond what is currently being taught in most entry-level doctor of physical therapy curricula. Given the broad applicability of motor learning to all areas of physical therapy, this progress warrants an update to the motor learning–related knowledge base of physical therapists. Providing newly trained physical therapists with a comprehensive understanding of motor learning research will promote the translation of up-to-date motor learning principles that can be used to guide clinical practice.

## Future Research Directions

As we begin the process of translating these scientific advances into clinical practice, it is important to be aware of the questions that remain unanswered. First, we do not yet know how to optimize long-lasting motor learning through sensorimotor adaptation or reinforcement learning mechanisms. Our foundational understanding of motor learning optimization (well-known principles of neuroplasticity and practice structure) was established primarily through studies of instructive and use-dependent learning. The degree to which these same optimization principles translate to sensorimotor adaptation or reinforcement learning is currently unknown. The results of a recent study^76^ suggest that the same “rules of thumb” may not apply to sensorimotor adaptation, highlighting the need for additional research to fully understand how we might optimize learning through each of these mechanisms.^77^

**Figure 3:**
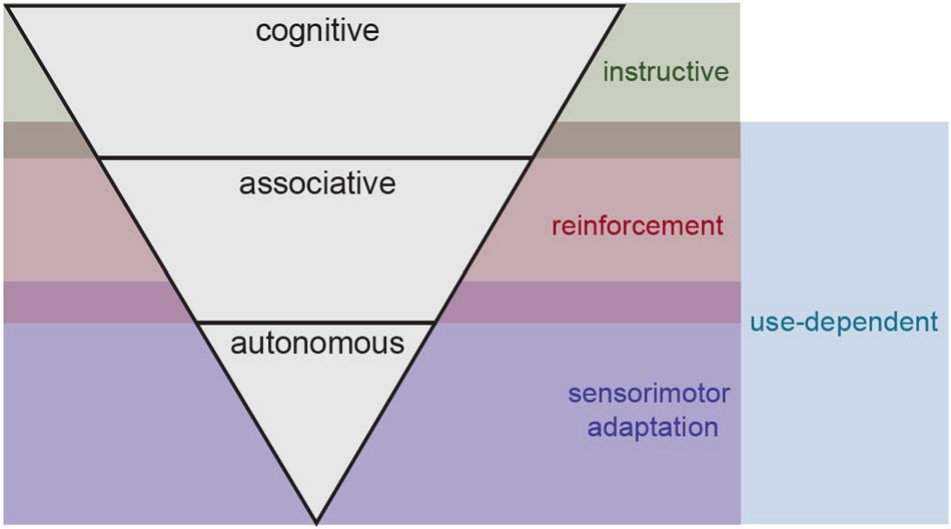
A proposed contribution of use-dependent, instructive, reinforcement, and sensorimotor adaptation motor learning mechanisms to the stages of motor learning described by Fitts and Posner.^34^

Future research is also needed to elucidate how different learning mechanisms interact during the course of learning a new motor behavior. As previously mentioned, even though we may be able to influence their relative contributions by carefully controlling practice parameters, multiple learning mechanisms are often engaged in parallel during a single learning experience. It is of particular importance to understand how the relative contributions of different mechanisms change over the course of learning. Considering the well-known stages of learning described by Fitts and Posner^34^ (Fig. 3), it is possible that instructive and reinforcement motor learning are more heavily engaged in the initial, cognitive stage of learning when performance is variable and the learner has to explore different movement solutions, whereas the associative stage engages primarily reinforcement and use-dependent mechanisms as the learner continues to refine their behavior with extensive practice. In the autonomous stage, further adjustments to motor behavior and a shift to automaticity may primarily be driven by sensorimotor adaptation and continued movement practice (similar to that suggested by the authors of previous reviews^21^^,^^40^). This working theory aligns with Gentile’s description of explicit and implicit processes that contribute to motor skill acquisition.^78^ Future research is needed to investigate these proposed weightings across the time course of learning because this information would allow physical therapists to strategically evolve their treatments to support different phases of learning over time. Improving our understanding of how each of these mechanisms contributes to motor learning over time also holds the potential to disentangle what is needed to facilitate the formation of a habitual motor behavior—the end goal of most movement rehabilitation.

Thus far, research about these motor learning mechanisms has primarily involved the study of upper extremity motor tasks. However, upper extremity, lower extremity, and whole-body movements are thought to be largely controlled by different components of the central nervous system (ie, cortical, spinal, propriospinal controllers, respectively). Therefore, there may be differences in how motor learning occurs and is promoted with each type of motor task. For example, massed reaching practice likely involves more cognitive processing than massed stepping practice. However, even with tasks like walking that are considered more automatic, the structure of an intervention can influence the amount of cognitive processing required (eg, gait training with obstacle negotiation or community mobility). Nonetheless, active engagement in the task is a baseline requirement to promote use-dependent learning during both reaching and walking. Future research is needed to test the efficacy of these different learning mechanisms to learn a wide range of motor tasks.

Perhaps the most limiting gap in our knowledge is that we do not yet know how these motor learning mechanisms can be harnessed to address limitations in activity or participation. The majority of motor learning studies, even those conducted with clinical populations, have been designed to evaluate the effect of different motor learning mechanisms on changing specific movement kinematics. However, the link between kinematic changes and functional improvement is not clear. Therefore, further research is needed to determine how each of these distinct motor learning mechanisms can be effectively integrated into the design of functional mobility interventions.

Finally, it is important to note that studies focused on the translation of these advances in motor learning research into clinically applicable treatment approaches are largely preliminary. To date, few studies have evaluated the effects of long-term interventions that are primarily underpinned by instructive, reinforcement, or sensorimotor adaptation-based motor learning. Whereas a number of studies have tested the effects of multiday training protocols in neurotypical and patient populations, very few studies have evaluated longer-term interventions that are designed to intentionally harness these motor learning mechanisms. Future work must expand upon the large body of work that demonstrates the efficacy of use-dependent learning-based physical therapist interventions and (1) determine how to translate our knowledge of these other motor learning mechanisms into clinically applicable interventions, and (2) assess the effects of these interventions on motor behaviors of interest.

## Conclusions

The concepts described here denote an expansion of the scientific theory related to human motor learning. We now appreciate that motor learning occurs through multiple distinct mechanisms and neural processes. A more comprehensive understanding of motor learning has many practical implications for physical therapy and movement rehabilitation. It is critical that we integrate this information into our working knowledge of motor learning. Doing so will enrich our understanding of the science that motivates our interventions and is likely to stimulate clinical and research innovation. This process—evolution of thought and behavior based on new information—is essential for continued evidence-based practice in physical therapy.
